# Oligo swapping method for in vitro DNA repair substrate containing a single DNA lesion at a specific site

**DOI:** 10.1186/s41021-018-0112-5

**Published:** 2018-11-12

**Authors:** Mika Yukutake, Mika Hayashida, Narumi Shioi Aoki, Isao Kuraoka

**Affiliations:** Department of Chemistry, Faculty of Science, Fukuoka University, 8-19-1 Nanakuma, Jonan-ku, Fukuoka, 814-0180 Japan

**Keywords:** DNA lesion, In vitro DNA repair substrate, In vitro assay

## Abstract

**Background:**

A wide variety of DNA lesions interfere with replication and transcription, leading to mutations and cell death. DNA repair mechanisms act upon these DNA lesions present in the genomic DNA. To investigate a DNA repair mechanism elaborately, an in vitro DNA repair substrate containing DNA lesions at a specific site is required. Previously, to prepare the substrate, phagemid ssDNA and DNA lesion-harboring oligonucleotides were employed with considerable amounts of DNA polymerase and DNA ligase. However, preparing in vitro DNA repair substrate in general is difficult and labor intensive.

**Results:**

Here, we modified the construction method of in vitro mismatch repair substrate using a nicking-endonuclease, which produces gap corresponding to the ssDNA in the plasmid DNA, and swaps DNA lesion-containing oligonucleotide upon addition of restriction enzyme and T5 exonuclease. This modified method is able to produce in vitro DNA repair substrates containing adenine:cytosine mismatch basepair, 8-oxoG, and uracil. The DNA repair enzyme, each Fpg, hOGG1 could cleave an 8-oxoG-containing DNA substrate, the mixture of UDG and APE1 could cleave a uracil-containing DNA substrate. Omitting a column purification step, DNA repair substrates were prepared by one-pot synthesis.

**Conclusions:**

We were able to prepare in vitro DNA repair substrates using this simple method involving restriction enzymes and T5 exonuclease. It is anticipated that this method, termed as “Oligo Swapping Method”, will be valuable for understanding the DNA repair machinery.

## Background

Although genomic DNA supposedly contains error-free genetic information to facilitate the proper cell functioning, yet it is prone to damage, deterioration, and modifications due to environmental and endogenous factors. The induced DNA lesions interfere with DNA and RNA synthesis during replication and transcription, respectively, leading to mutations and cell death [1]. As a result, these genomic mutations and subsequently, the disruption of cellular processes may cause cancer, congenital diseases, and aging. To maintain genomic integrity, cells possess several DNA repair pathways such as nucleotide excision repair (NER), which operates primarily on genotoxic chemicals or UV irradiation-induced bulky helix-distortion (e.g. CPD, 6–4 pp),; base excision repair (BER) (short- and long-patch repairs), which is used for non-bulky and non-helix-distorting DNA modifications induced by alkylation, oxidation (e.g. 8-oxoG), and deamination (e.g. uracil); and mismatch repair (MMR), which mends the mismatch base pairings during DNA replication. DNA repair systems are highly conserved from bacteria to humans [2, 3].

To analyze DNA repair mechanisms in vitro, we required DNA repair substrates containing a DNA lesion at specific site. For the in vitro BER assay for short patch repair, chemically synthesized DNA lesion bearing oligonucleotides annealed with complementary strands were employed as DNA lesion substrates [4, 5]. Because DNA glycosylases of BER enzymes can digest a short oligonucleotide substrate (10–30-mer) containing a DNA lesion, it is not difficult to prepare DNA substrates for incision reaction during BER. On the other hand, in case of in vitro NER assay [6, 7], at least ~ 120-bp long DNA substrates for the reaction are required [8]. In addition, in vitro long patch BER [9] is also required to allow the repair action of DNA polymerase and proliferating cell nuclear antigen (PCNA) on the plasmid to fill in the gap [10]. Thus, the DNA substrates containing lesions were produced by in vitro DNA synthesis using single strand plasmid DNA annealed chemically with synthesized lesions-bearing oligonucleotide and DNA polymerase and ligase. Moreover, to observe DNA repair during NER and BER, covalently closed circular DNA substrate is required because the repair systems do not act upon linear DNA substrate. Additionally, for MMR assay, the DNA mismatch substrates were produced by a hybridization reaction between a circularized ssDNA and a digested plasmid DNA [11]. Although these methods are useful, they carry limitations like difficulty in preparation and high expenditure.

In vitro DNA MMR substrate construction method is a reasonable one to induce the DNA lesion at the specific site. In this method, a nicking endonuclease produces a ssDNA stretch on a purified dsDNA plasmid for replacement by synthetic oligonucleotide and thereafter, DNA ligase seals the nick site. This was reported by Wang et al. reported in 2000 and 2002 [12, 13]. Later, Du et al. reported the procedure in 2011 [14] with modifications, i.e. adding a purification step of ssDNA by using the streptavidin linked oligonucleotides. Although these methods are used for MMR assays, they can also be used potentially to induce DNA lesions in plasmid DNA.

Here, we have modified the method by addition of a restriction enzyme and T5 exonuclease that is capable of removing linear dsDNA and nicked plasmid DNA. By the Oligo Swapping Method, we produced in vitro DNA repair substrates containing adenine:cytosine (A:C) mismatch, 8-oxoG, and uracil residues at specific sites. This method allows execution of one-pot reaction without any DNA purification steps during the substrate production.

## Methods

### Enzymes and chemicals

Nt.*Bbv*CI nicking endonuclease, *Eco*NI, Fpg, hOGG1, UDG, APE1, and T5 exonuclease were purchased from New England Biolabs (Ipswich, MA, USA). *Kpn*I, *Sac*I, and T4 DNA ligase were from TaKaRa (Otsu, Shiga, Japan). Enzyme reactions (10 μL) for DNA repair assay were carried out according to the manufacturer’s instructions and were terminated by adding 2 μL Gel Loading Dye, Purpule from New England Biolabs.

### Oligonucleotides

For construction of pBS2-SDL, 5′-CCCGGTACCCCCGAATTCGCCTCAGCCCTAATCGAGGCGTTTCCCTCAGCGGCTGCAGCACGAGCTCCCC-3′ and 5′-GGGGAGCTCGTGCTGCAGCCGCTGAGGGAAACGCCTCGATTAGGGCTGAGGCGAATTCGGGGGTACCGGG-3′ were synthesized and purified with cartridge at FASMAC (Kanagawa, Japan). Both oligonucleotides were hybridized and digested with *Kpn*I and *Sac*I to produce synthetic 64-bp DNA duplex. The products were purified by PCR Clean-up kit (MACHEREY-NAGEL, Düren, Germany). For synthesis of the DNA substrate plasmid which contains single DNA lesions, mismatch oligos (5′-p-TCAGCCCTAATCGAAGCGTTTCCC-3′; A is mismatch base; p is phosphate), 8-oxoG oligos (5′-p-TCAGCCCTAATCGA8GCGTTTCCC-3′; 8 is 8-oxoG; p is phosphate), and uracil oligos (5′-p-TCAGCCCTAATCGAUGCGTTTCCC-3′; U is uracil; p is phosphate) were synthesized and purified by HPLC at FASMAC.

### Construction of pBS2-SDL

**Fig. 1:**
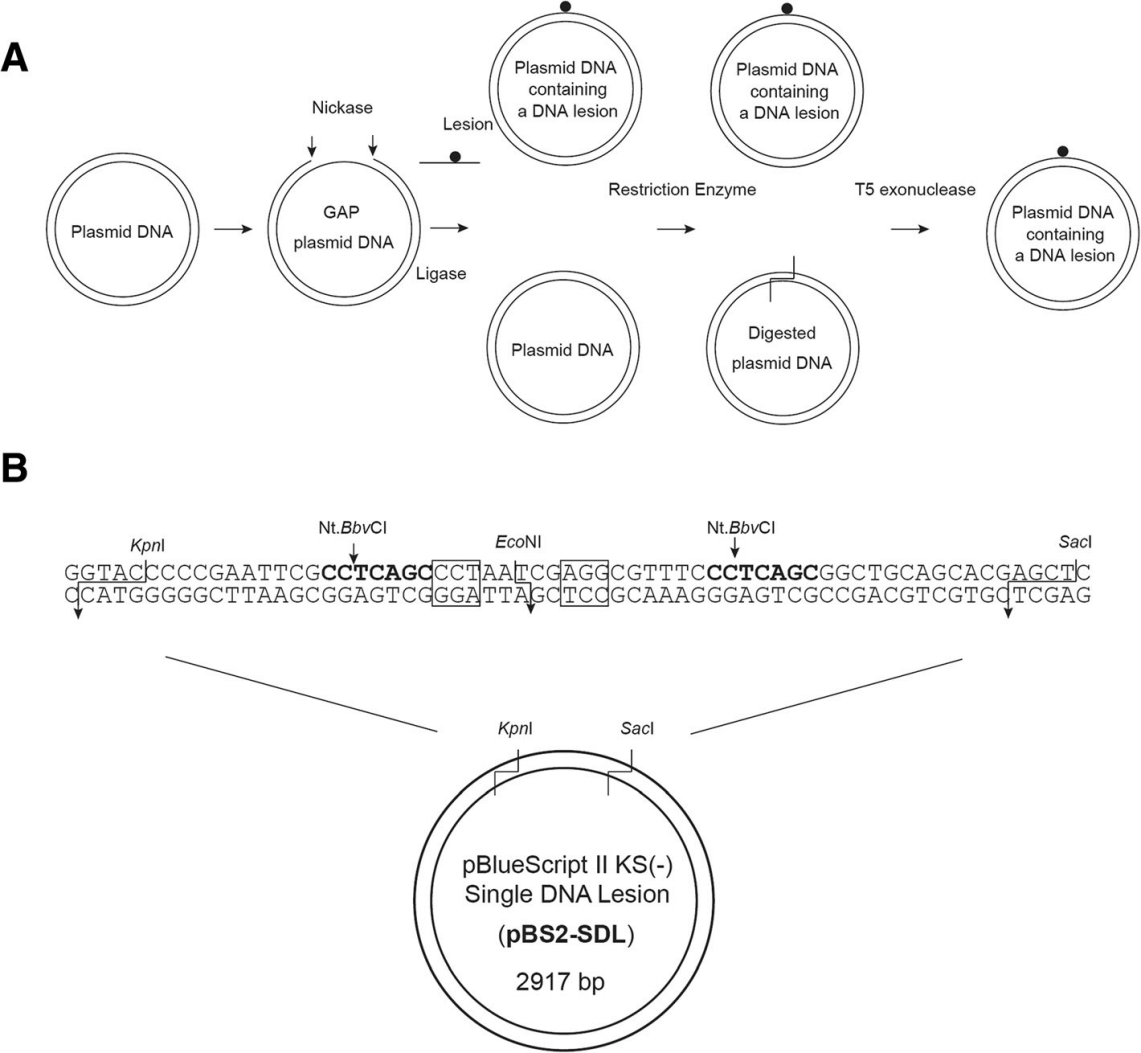
**a** Experimental design. The plasmid pBS2-SDL was digested with a nicking endonuclease. An oligonucleotide containing a DNA lesion was hybridized with gap plasmid and ligated using T4 DNA ligase. Original plasmids in the sample are digested with restriction enzymes, except for DNA lesion bearing plasmids. T5 exonuclease cuts only the linear DNA plasmids digested by *Eco*NI, and does not work on sealed DNA plasmids containing a DNA lesion. **b** Covalently closed circular duplex DNA containing a single lesion. Sixty four-basepair oligonucleotides containing a single DNA lesion site within the *Eco*NI restriction enzyme site, two nicking endonuclease sites and the plasmid pBS2-SDL (2917 bp) are shown diagrammatically

The pBlueScript II KS (−) Single DNA Lesion (pBS2-SDL) plasmid was constructed by replacing the *Kpn*I-*Sac*I fragment of pBlueScript II KS (−) with the synthetic 64-bp DNA duplex containing the target site for nickase Nt.*Bbv*CI, and the restriction enzyme *Eco*NI (Fig. 1b). Plasmid DNAs were purified by NucleoSpin Plasmid. The construct pBS2-SDL was checked by DNA sequencing.

### Standard procedure for synthesis of in vitro DNA repair substrate

Nicking reaction mixture (20 μl) was comprised of pBS2-SDL (3 pmol) and Nt.*Bbv*CI (5 units) in reaction buffer containing 50 mM Potassium acetate, 20 mM Tris-acetate (pH 7.9), 10 mM Magnesium acetate, and 100 μg/ml bovine serum albumin. The reactions were incubated at 37 °C for 3 h and then at 80 °C for 20 min. They were then purified using PCR Clean-up kit (MACHEREY-NAGEL GmbH & Co.) to remove the 24-mer oligonucleotides digested by Nt.*Bbv*CI. After purification, the DNA sample mixture (about 25 μl) prepared in the same reaction buffer was incubated with chemically synthetized 24-mer DNA lesion oligonucleotide (15 pmol) at 95 °C for 5 min, 70 °C for 5 min and RT for 15 min for an annealing reaction. For DNA ligation reactions, the mixtures were incubated overnight at 16 °C in the presence of ATP (final concentration 1 mM) and T4 DNA ligase (800 units) in 30 μl system, and the reaction was terminated by incubating at 65 °C for 10 min. For restriction endonuclease *Eco*NI reaction, *Eco*NI (5 units) was added to the reaction sample (30 μl) followed by incubation of the mixtures at 37 °C for 1 h and at 65 °C for 20 min. For T5 exonuclease reaction, the mixtures were incubated with T5 exonuclease (5 units) at 37 °C for 1 h. For DNA purification step, T5 exonuclease treated mixtures were purified by PCR Clean-up kit (MACHEREY-NAGEL). For one-pot synthesis, first DNA purification step was omitted.

### Agarose gel electrophoresis

The sample DNA substrates were subjected to 0.8% agarose gel, and visualized by ethidium bromide staining. DNA fragments in the agarose gel were analyzed using ImageJ software. % contamination = (Open circular DNA + liner DNA / (Open circular DNA + liner DNA + covalently closed circular DNA) × 100.

## Results & discussion

An experimental design has been shown in Fig. 1a. The plasmid DNA, pBluescript II KS(−) Single DNA lesion (pBS2-SDL), containing two nicking sites for endonuclease Nt.*Bbv*CI and restriction endonuclease *Eco*NI, (Fig. 1b) was digested by Nt.*Bbv*CI to produce 24-mer gap region of ssDNA on the plasmid. The digested oligonucleotides were then removed from the plasmid by a DNA purification step, followed by addition of the oligonucleotide containing DNA lesion. After ligation, the plasmid DNA was digested by *Eco*NI to remove plasmid containing residual non-lesion oligonucleotides. The reaction mixtures were digested by T5 exonuclease that is capable of removing linear dsDNA and nicked plasmid DNA, but not supercoiled plasmid DNA [15, 16]. This method allows production of site-specific lesion-bearing DNA substrates.

**Fig. 2:**
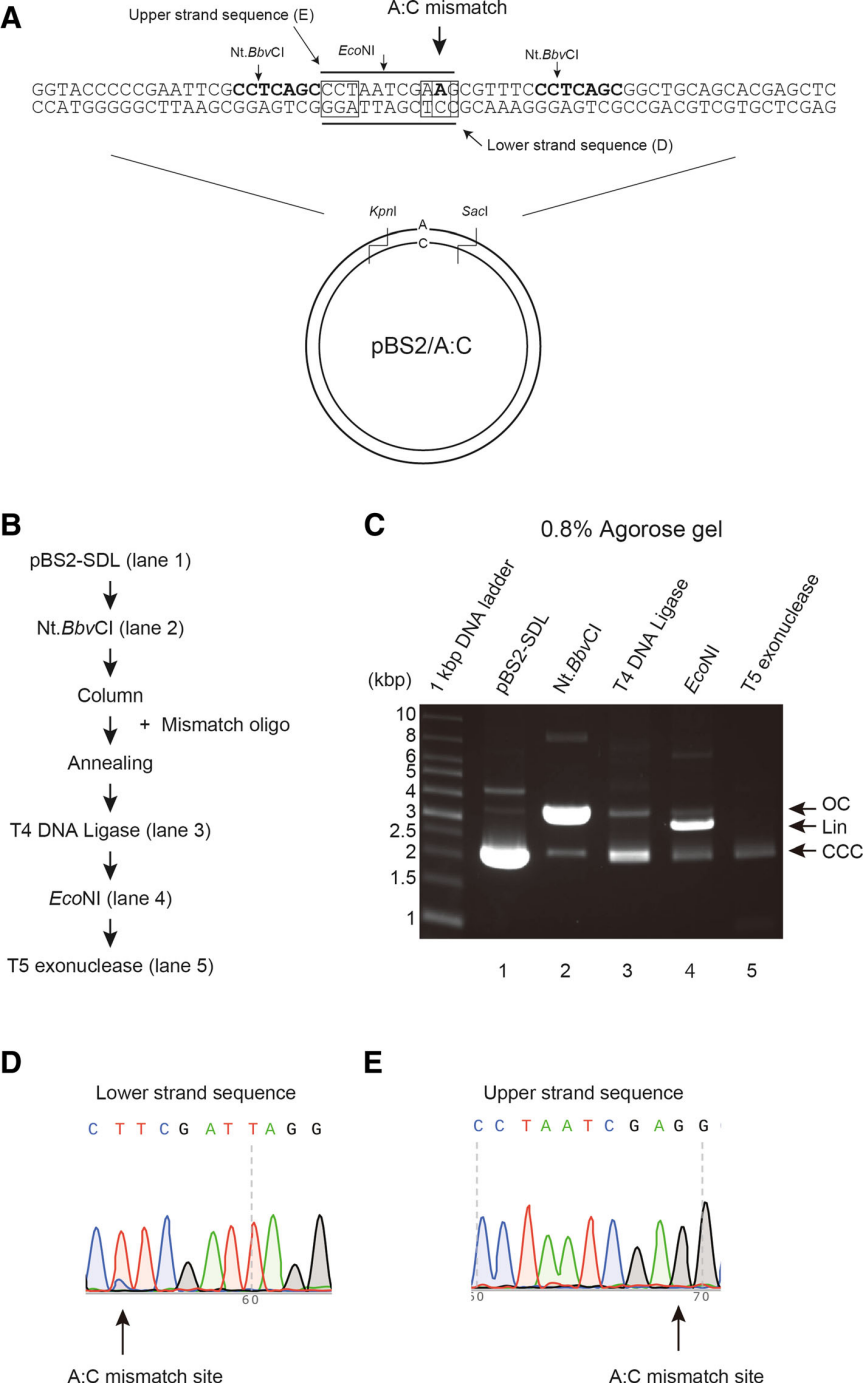
Preparation of DNA template (A:C mismatch substrate) with A:C mismatch at a defined position. **a** Upper strand sequence containing A base and lower strand substrate containing original C base in pBS2/A:C are shown diagrammatically. **b** Experimental procedure for purification of pBS2/A:C. **c** Aliquots from various steps of the purification were analyzed on 0.8% agarose gel, and the DNA substrates were visualized by staining with EtBr. Lane 1, pBS2-SDL; lane 2, Nt.BbvCI-treatment; lane 3, T4 DNA ligase-treatment; lane 4, *Eco*NI-treatment; lane 5, T5 exonuclease-treatment. Lower panel shows final purified DNA products (%). Open circular DNA (OC), linear DNA (Lin), and covalently closed circular DNA (CCC) are indicated by arrow. **d** Upper strand sequence containing A base in pBS2/A:C was sequenced. An A:C mismatch site is indicated by the arrow. **e** Lower strand sequence containing base A in pBS2/A:C was sequenced. The A:C mismatch site is indicated by the arrow

We tested the efficacy of this method in producing A:C mismatch substrates (Fig. 2a) using mismatch oligonucleotide. Figure 2b shows experimental procedure for purification of pBS2/A:C. Purified pBS2-SDL (Fig. 2c, line 2) was digested by Nt.*Bbv*CI to produce the 24-mer gap and was purified to remove Nt.*Bbv*CI-digested oligonucleotides (Fig. 2c, line 3). The mixture was incubated with T4 DNA ligase at 16 °C overnight (Fig. 2c, line 4), followed by *Eco*NI digestion (Fig. 2c, line 5). The mixture was finally treated with T5 exonuclease and purified by DNA purification step (Fig. 2c, line 6). Post purification, pBS2/A:C DNA substrate was observed as one single band on 0.8% agarose gel.

To confirm the presence of A:C mismatch in this DNA substrate, each of the purified plasmid DNA strands was sequenced. While the upper strand sequence showed mainly T and a little C at the mismatch position, the lower strand sequence showed only G, indicating that this method can produce A:C mismatch on the DNA substrate. We observed slight contamination with the original plasmid.

**Fig. 3:**
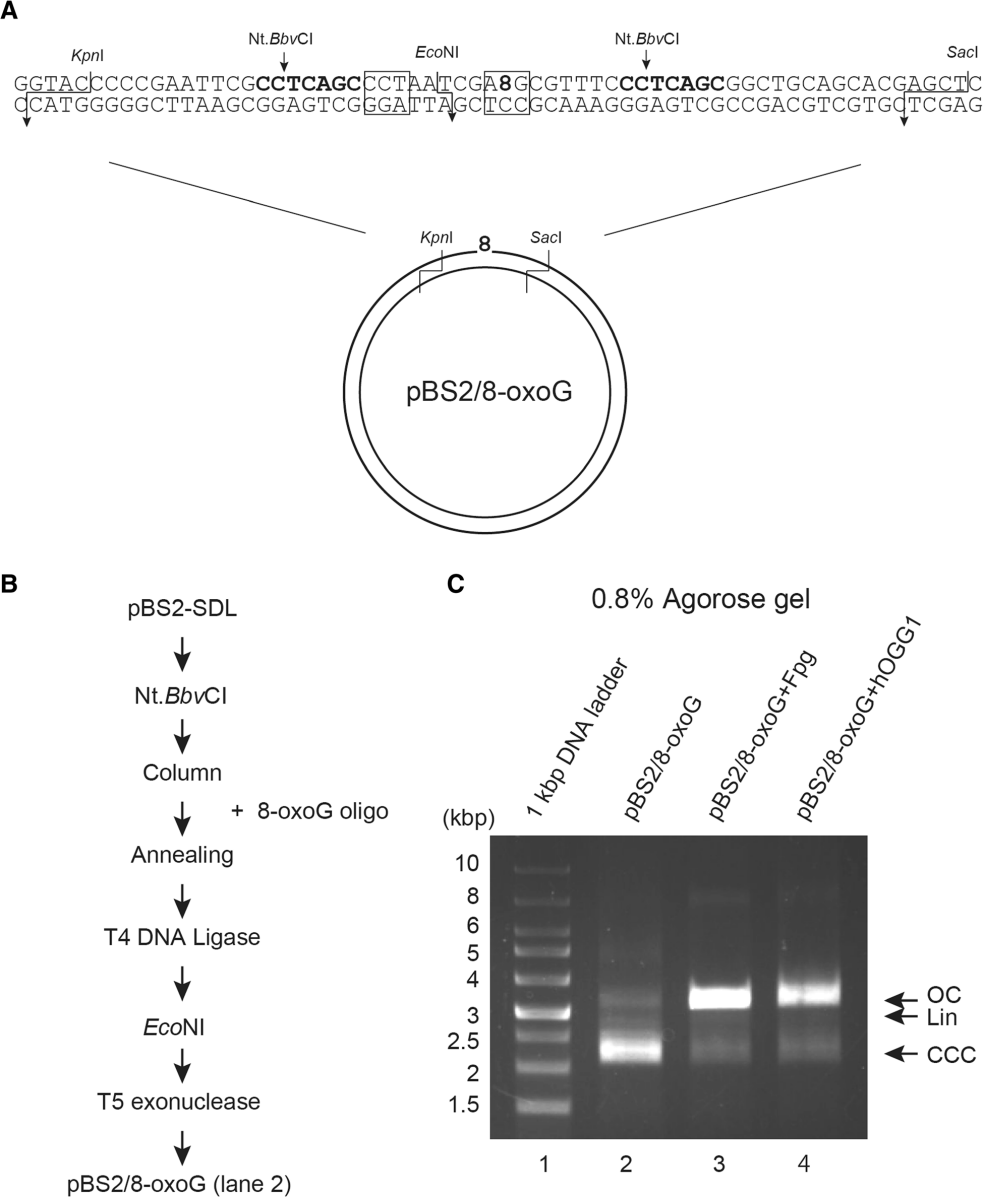
Preparation of DNA template (8-oxoG substrate) with 8-oxoG at a defined position. **a** Upper strand sequence containing 8-oxoG base and lower strand substrate containing original C base in pBS2/8-oxoG are shown diagrammatically. **b** Experimental procedure for purification of pBS2/8-oxoG using 8-oxoG oligo. **c** Identified DNA substrates (100 ng), pBS2/8-oxoG, were incubated with Fpg (2 units) or hOGG1 (0.16 units) at 37 °C for 30 min. Aliquots from the sample were run on 0.8% agarose gel and visualized by staining with EtBr. Lane 1, 1-kbp marker; lane 2, non-treatment; lane 3, Fpg-treatment; lane 4, hOGGI-treatment. Open circular DNA (OC), liner DNA (Lin), and covalently closed circular DNA (CCC) are indicated by arrows

Next, we tried to introduce an oxidative DNA lesion 8-oxoG in the plasmid DNA using this method. 8-oxoG was located within/at the *Eco*NI recognition site of the oligonucleotide, causing inhibition of *Eco*NI mediated digestion (Fig. 3a). Figure 3b shows the experimental steps followed for the purification of pBS2/8-oxoG. To confirm if this in vitro DNA repair substrate contained 8-oxoG, the substrates purified by this method were treated with Fpg [17, 18] or hOGG1 [19]. The two DNA repair enzymes act as both an N-glycosylase and an AP-lyase. The N-glycosylase activity releases damaged purines from double stranded DNA, generating an AP site. The AP-lyase activity cleaves both at 3′ and 5′ ends of the AP site, thereby removing it and leaving a single-base gap. When pBS2/8-oxoG DNA substrates were incubated with these enzymes, open circular conformation of plasmid DNA substrate was observed on 0.8% agarose gel, whereas linear DNA was not seen (Fig. 3c, lanes 3 and 4). In addition, most of the supercoiled DNA substrates converted to open circular form, indicating the presence of 8-oxoG lesion in this DNA substrate.

**Fig. 4:**
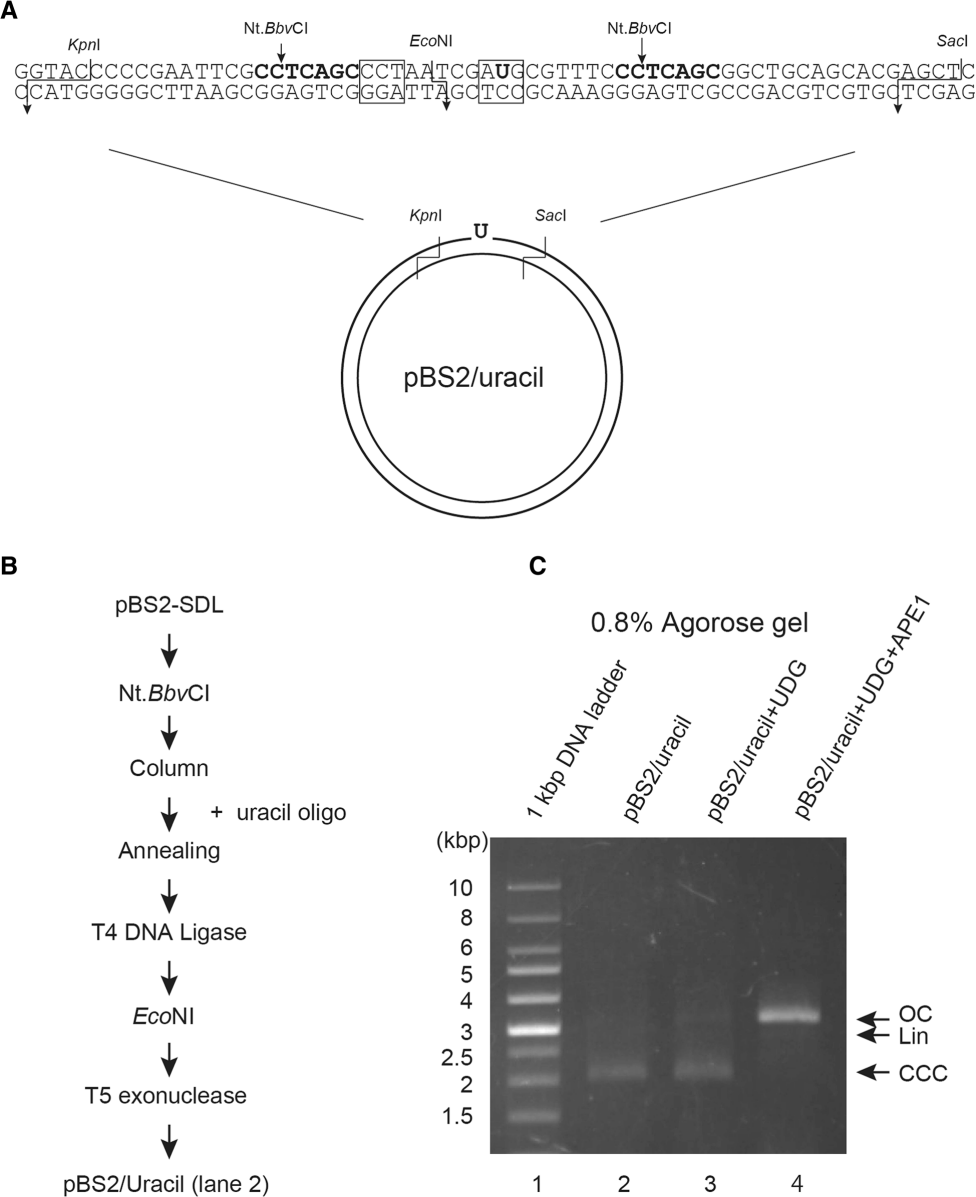
Preparation of DNA template (uracil substrate) with uracil at a defined position. **a** Upper strand sequence containing uracil base and lower strand substrate containing original C base in pBS2/Uracil are shown diagrammatically. **b** Experimental procedure for purification of pBS2/uracil using uracil oligo. **c** Identified DNA substrates (100 ng), pBS2/uracil, were incubated with UDG (0.5 units) or UDG (0.5 units) + APE1 (5 units) at 37 °C for 30 min. Aliquots from the sample were analyzed on 0.8% agarose gel, and the DNA substrates were visualized by staining with EtBr. Lane 1, 1-kbp marker; lane 2, non-treatment; lane 3, UDG-treatment; lane 4, UDG + APE1-treatment. Open circular DNA (OC), liner DNA (Lin), and covalently closed circular DNA (CCC) are indicated by arrows

For further applications of this method, we introduced a deaminated uracil as the DNA lesion on the plasmid DNA. Uracil was positioned at the *Eco*NI recognition site of the oligonucleotide to inhibit *Eco*NI digestion (Fig. 4a). Figure 4b shows the experimental procedure for purification of pBS2/uracil. Again, to confirm whether this in vitro DNA repair substrate contained uracil, the purified substrate was treated with UDG [20] and then APE1 [21, 22]. UDG catalyzes the release of free uracil from uracil-containing DNA, producing the AP site. Thus, upon incubation with UDG, no change in migratory behavior of pBS2/uracil was observed on agarose gel (Fig. 4c, lane 3). When UDG and APE1 were incubated with the uracil containing DNA substrates, most of the supercoiled DNA substrates transformed into open circular plasmids (Fig. 4c, lane 4), suggesting that this method is able to produce uracil lesion within the plasmid DNA.

**Fig. 5:**
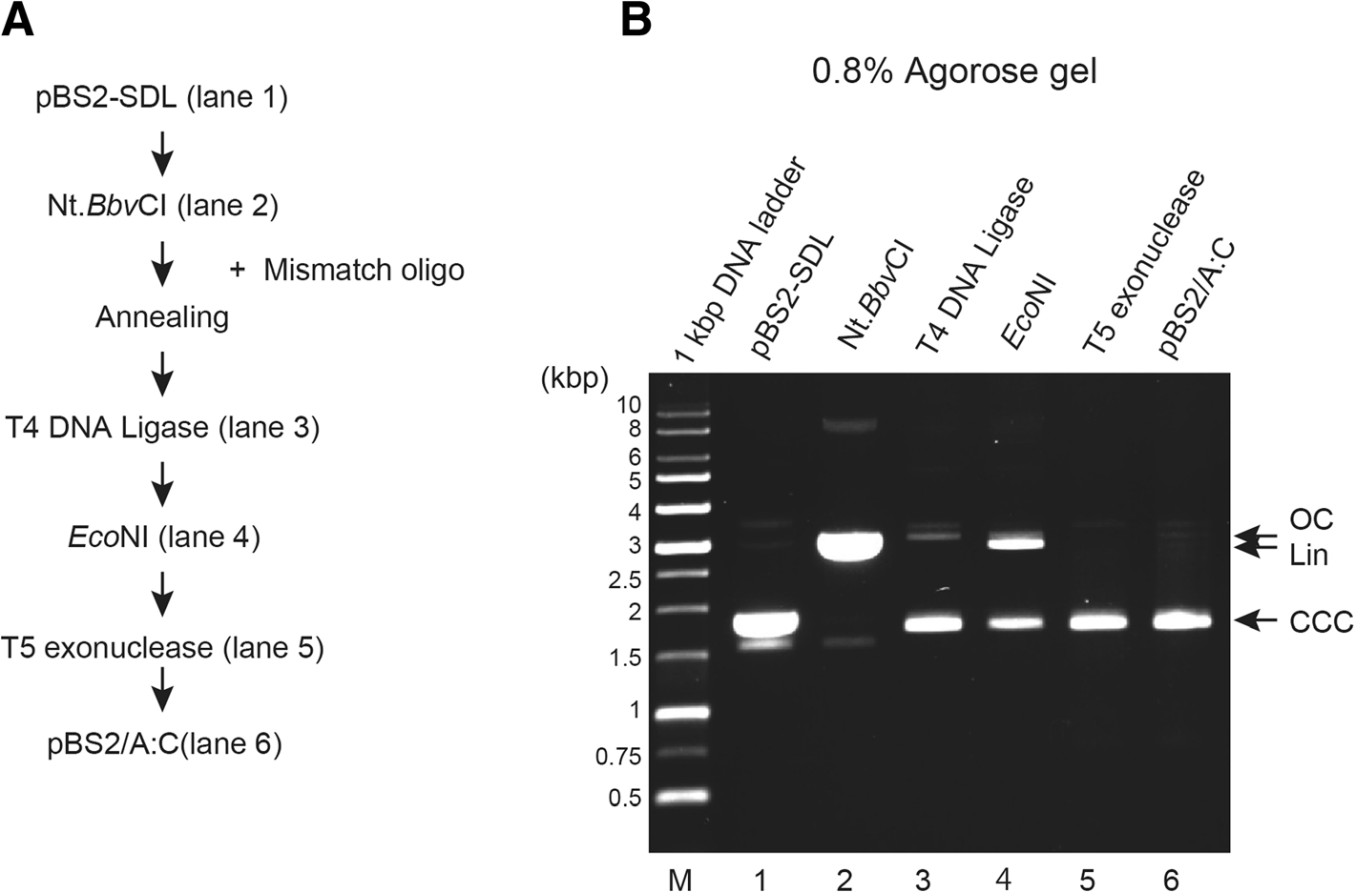
One-pot synthesis of DNA repair substrate. **a** Experimental procedure for purification of pBS2/A:C omitting a column purification step. **b** Aliquots from various steps of the purification were subjected to 0.8% agarose gel electrophoresis, and the DNA substrates were visualized by staining with EtBr. Lane 1, pBS2-SDL; lane 2, Nt.BbvCI-treatment; lane 3, T4 DNA ligase-treatment; lane 4, *Eco*NI-treatment; lane 5, T5 exonuclease-treatment: lane 6, purified pBS2A:C by PCR purification kit. Open circular DNA (OC), liner DNA (Lin), and covalently closed circular DNA (CCC) are indicated by arrows. And the irreversibly denatured form was observed as the minor band shorter than the CCC band.

We used this method by recruiting restriction endonuclease and T5 exonuclease to induce site-specific DNA lesion in the plasmid. To simplify this method further, we attempted to synthesize the DNA substrate in one 1.5 ml microcentrifugation tube. Figure 5a, when compared with Fig. 2b, shows the experimental procedure for purification of pBS2/A:C omitting a DNA purification step. When this procedure was executed, population with incorporation of a mismatched oligonucleotide seemed to be better with respect to the procedure involving the additional DNA purification step (compare Fig. 2c, lane 5 and Fig. 5b, lane 4). Moreover, in the method containing the DNA purification step, 81.1% of products were digested by *Eco*NI, indicating 81.1% contamination, while in case of one-pot synthesis, only 53.6% of products were digested by *Eco*NI. Some reagents in the DNA purification step might interfere with the following reactions for the DNA synthesis. Thus, one-pot synthesis worked more efficiently under our experimental conditions. Therefore, we have successfully established the one-pot synthesis protocol for in vitro DNA repair substrate containing site-specific DNA lesion.

## Conclusions

To analyze DNA repair mechanisms in vitro, DNA substrates having DNA lesions at particular sites are necessary. There are several procedures to produce the DNA lesion bearing substrate. In some cases, however, it is not easy to prepare the DNA substrate and proves to be expensive. Here we establish the procedure for preparing DNA substrate containing site-specific DNA lesion. This modified method using restriction enzyme and T5 exonuclease allows the preparation of the DNA substrate on which the DNA repair enzymes function. Moreover, one-pot synthesis method makes it more simple and convenient to prepare the DNA substrate. We believe that this method is useful to investigate DNA repair mechanisms in vitro.

## Abbreviations

6–4 pp.: pyrimidine (6–4) pyrimidone photoproduct
8-oxoG: 8-oxoguanine
AP: Apurinic/apyrimidinic site
APE1: Apurinic/apyrimidinic endonuclease
BER: Base excision repair
CPD: Cis-syn cyclobutane pyrimidine dimer
dsDNA: Double strand DNA
EtBr: Ethidium bromide
Fpg: Formamidopyrimidine [fapy]-DNA glycosylase
hOGG1: human 8-oxoguanine DNA glycosylase
MMR: Mismatch repair
NER: Nucleotide excision repair
PCNA: Proliferating cell nuclear antigen
ssDNA: single strand DNA
UDG: Uracil-DNA Glycosylase
